# Human CYP2C9 Metabolism of Organophosphorus Pesticides and Nerve Agent Surrogates

**DOI:** 10.3390/jox16010001

**Published:** 2025-12-19

**Authors:** Pratik Shriwas, Abigail M. Noonchester, Andre Revnew, Thomas R. Lane, Christopher M. Hadad, Sean Ekins, Craig A. McElroy

**Affiliations:** 1 Division of Medical Chemistry and Pharmacognosy, College of Pharmacy, The Ohio State University, Columbus, OH 43210, USA; 2 Department of Chemistry and Biochemistry, College of Arts and Sciences, The Ohio State University, Columbus, OH 43210, USAhadad.1@osu.edu (C.M.H.); 3 Collaborations Pharmaceuticals, Raleigh, NC 27606, USA

**Keywords:** CYP2C9, LC-MS/MS, organophosphorus pesticides, high-throughput screening, toxicity, metabolism

## Abstract

Of the Cytochrome P450 enzymes, the CYP2C9 variant is very important in the metabolism of several human drugs, acting as a natural bioscavenger. Previously, CYP2C9 was shown to convert the thion (P=S) to the oxon (P=O) form for some organophosphorus (OP) pesticides, such as dimethoate, diazinon, and parathion. In this study, we tested the ability of CYP2C9 to degrade other OP compounds. We investigated the metabolism of OP compounds by CYP2C9 using LC-MS/MS as well as time-dependent inhibition using the previously developed pFluor50 fluorogenic assay. We found that CYP2C9 metabolizes thions preferentially over oxons, and that many OP compounds inhibit CYP2C9 activity in a time-dependent manner. Additionally, we performed molecular docking based on the crystal structure (1OG5) of the CYP2C9 receptor. We observed a positive, though moderate, correlation between the calculated binding energy and the CYP2C9 metabolism of various OP compounds (R = 0.59). These in vitro data, combined with further analysis and additional OP derivatives, could potentially be used to develop artificial intelligence (AI)/machine learning (ML) models to predict the metabolism of specific OP compounds by CYP2C9. This type of approach could be particularly relevant for the prediction of the metabolism of current and emerging chemical warfare agents.

## 1. Introduction

Exposure to organophosphorus (OP) compounds results in both acute and chronic symptoms due to covalent inhibition of acetylcholinesterase (AChE) [1]. Deliberate OP pesticide exposure results in a very high number of suicide fatalities, with approximately 150,000–200,000 deaths recorded every year worldwide [2,3], representing ~15% of all suicidal deaths per year [4]. Global pesticide use continues to increase, with an estimated 3.7 million metric tons used per year in 2022 [5], leading to as many as 25 million agricultural workers worldwide experiencing unintentional pesticide poisoning each year [6], with significant health consequences, such as cancer and neurotoxicity [7]. Additionally, the residual pesticide that remains on the crops, such as fruits and vegetables [8,9,10], leads to chronic OP exposure for a significant percentage of the population. More intentional OP poisoning comes from another class, chemical warfare nerve agents (CWNAs), wherein attacks on individuals or groups of people often result in death due to their high toxicity. The highest-profile tragedies include the use of sarin in Syria in 2013 [11], the assassination of Kim Jong Nam in Kuala Lumpur with VX in 2017 [12], the use of a Novichok agent in an assassination attempt on Yulia and Sergei Skripal, resulting in the death of Dawn Sturgess in Salisbury UK in 2018, and, most recently, in the poisoning (2020) of Alexei Navalny in Russia [13].

OP compounds can be divided into two major groups, oxons (P=O) or thions (P=S), depending on whether the phosphorus atom is bonded to oxygen or sulfur, respectively [14]. Chemical warfare nerve agents are oxons, while OP pesticides can be delivered directly as oxons or as thions, which are then metabolized to the oxon form, thereby creating the active metabolite that inhibits the critical enzyme, AChE [15]. Currently, the treatment for OP poisoning includes the use of the anticholinergic agent atropine and the use of AChE reactivators, such as pralidoxime, obidoxime, or HI-6, each of which has some efficacy [16,17]. However, due to inefficient crossing of the blood–brain barrier, these pyridinium oxime reactivators are not effective in the central nervous system (CNS). Quinone methide precursors mitigate many of the weaknesses of pyridinium oximes as reactivators of OP-inhibited AChE, while providing protection in the central nervous system; however, at present, such novel reactivators are still in development [18], and so there is currently no adequate therapy that has been approved for the treatment of OP poisoning in the CNS.

Hepatic cytochrome P450 (CYP) enzymes play a critical role in phase I metabolism of xenobiotics, including pharmaceuticals, and their inhibition is responsible for many drug–drug interactions [1]. Recently, we showed that human liver microsomes (HLMs) are capable of degrading many OP pesticides and CWNA surrogates using targeted NMR and LC-MS/MS metabolomics [19]. However, human HLMs consist of numerous enzymes, including a number of CYP enzymes, esterases, and amidases [20], so at present, it is unclear which specific enzymes are responsible for the metabolism of a given molecule.

In this study, we explored whether CYP2C9 was capable of metabolizing OP pesticides and CWNA surrogates using targeted LC-MS/MS metabolomics. Indeed, CYP2C9 is the most abundant member of the CYP2C family and contributes approximately 20% of the hepatic CYP content in HLMs [21,22]. It is also responsible for the metabolism of approximately 15% or around 100 clinical drugs [23]. CYP2C9 has previously been shown to desulfurate thion OPs, such as diazinon [24], dimethoate [25], disulfoton [26], fenthion [27], malathion [28], and ethyl parathion [29].

In this work, we found that CYP2C9 was more selective toward thions compared with oxons using LC-MS/MS analysis. Nonetheless, the CWNA surrogate oxons, specifically CMP (a cyclosarin surrogate) and PiMP (a soman surrogate), were metabolized by CYP2C9 (see Figures S1 and S2 in the Supporting Information for the structures). It was also determined that the CYP2C9 inhibition potency changed with both dose and time for nearly all OP compounds. We also performed in silico docking of the OP compounds to a CYP2C9 crystal structure to investigate the correlation between LC-MS/MS metabolism and the computed binding energy (BE) with R = 0.59 (*p* < 0.001). Such in vitro data, combined with additional analysis (like multiple linear regression and MD simulations), could potentially be used to develop an artificial intelligence/machine learning (AI/ML) model for the prediction of metabolism of OP compounds by CYP enzymes. This is of particular importance as new OP threats are always emerging and will likely be aided by the use of AI/ML, as demonstrated by the recent AI/ML-based prediction of 40,000 new OP structures (with many predicted to be more toxic) from the CWNA VX [30,31].

## 2. Materials and Methods

### 2.1. Chemicals

All OP pesticides were purchased in neat form and used without purification. Phosfolan (CAS: 947-02-4) and tetrachlorvinphos (TCVP, CAS: 22248-79-9) were purchased from AccuStandard, Inc. (New Haven, CT, USA). Bensulide (BEN, CAS: 741-58-2), chlorfenvinphos (CFVP, CAS: 470-90-6), chlorphoxim (CPH, CAS: 14816-20-7), chlorpyrifos oxon (CPO, CAS: 5598-15-2), crotoxyphos (CTP, CAS: 7700-17-6), crufomate (CFA, CAS: 299-86-5), cyanofenphos (CFP, CAS: 13067-93-1), diazinon (DIA, CAS: 333-41-5), dimefox (DME, CAS: 115-26-4), formothion (FMN, CAS: 2540-82-1), iodofenphos (CAS: 18181-70-9), isofenphos (IFP, CAS: 25311-71-1), isoxathion (IXT, CAS: 18854-01-8), methidathion (MDT, CAS: 950-37-8), mevinphos (MVP, CAS: 7786-34-7), phosalone (PHO, CAS: 2310-17-0), pyrazophos (PZP, CAS: 13457-18-6), schradan (SCN, CAS: 152-16-9), triazophos (TAP, CAS: 24017-47-8), and tribufos (TBS, CAS: 78-48-8) were purchased from LGC Limited (Manchester, NH, USA). Acephate (ACA, CAS: 30560-19-1), azinphos-methyl (APM, CAS: 86-50-0), chlorpyrifos (CPY, CAS: 2921-88-22), dichlorvos (DCV, CAS: 62-73-7), dimethoate (DMA, CAS: 60-51-5), ethoprophos (EPP, CAS: 13194-48-4), fenamiphos (FMP, CAS: 22224-92-6), fenthion (FNN, CAS: 55-38-9), iprobenfos (IBF, CAS: 26087-47-8), malathion (MAL, CAS: 121-75-5), mephosfolan (MPF, CAS: 950-10-7), methamidophos (MMP, CAS: 10265-92-6), monocrotophos (MCP, CAS: 6923-22-4), phosmet (PMT, CAS: 732-11-6), phosphamidon (PPM, CAS: 13171-21-6), primiphos-ethyl (PPE, CAS: 23505-41-1), pyridaphenthion (PPT, CAS: 119-12-0), quinalphos (QUI, CAS: 13593-03-8), temephos (TEM, CAS: 3383-96-8), and trichlorfon (TCP, CAS: 52-68-6) were purchased from Sigma-Aldrich (Milwaukee, WI, USA). Sulfaphenazole (CAS: 526-08-9), NADPH (CAS: 53-59-8), and MgCl_2_ (CAS: 7791-18-6) were purchased from Sigma-Aldrich and were HPLC-grade. The CWNA surrogates 3-cyano-4-methyl-2-oxo-2H-chromen-7-yl ethyl methylphosphonate (EMP, coumarin surrogate of VX), 3-cyano-4-methyl-2-oxo-2H-chromen-7-yl cyclohexyl methylphosphonate (CMP, coumarin surrogate of cyclosarin), 3-cyano-4-methyl-2-oxo-2H-chromen-7-yl (3,3-dimethylbutan-2-yl) methylphosphonate (PiMP, coumarin surrogate of soman), and ethyl (4-nitrophenyl) dimethylphosphoramidate (NEDPA, *p*-nitrophenol surrogate of tabun) were synthesized and generously supplied by Dr. Christopher Hadad and his research team at Ohio State University along with diisopropyl fluorophosphate (DFP, CAS: 55-91-4). The OP structures used in this study are presented as oxons or thions in Figures S1 and S2 (see the Supporting Information). All the OP derivatives containing *E*/*Z* or *R*/*S* conformations were used as a mixture of isomers. Supplementary Table S1 summarizes the general properties of OPs, including hydrogen bond donor (HBD), hydrogen bond acceptor (HBA), partition coefficient (logP), molecular weight (MW), rotatable bonds, polar surface area (PSA), and median lethal dose (LD_50_) [32,33,34,35,36,37,38,39,40].

### 2.2. pFluor50 CYP2C9 Inhibition Assay Using CypExpress™

CypExpress™ 2C9 (Sigma, MTOXCE2C9, St. Louis, MO, USA), containing full-length human recombinant CYP2C9, recombinant human P450 β-nicotinamide adenine dinucleotide phosphate (NADPH) oxidoreductase, and a proprietary mix of MgCl_2_, glucose-6-phosphate dehydrogenase, and glucose-6-phosphate, was purchased from Sigma Aldrich (St. Louis, MO, USA). Magnesium chloride and NADPH hydrate were purchased from Sigma Aldrich (St. Louis, MO, USA). The fluorogenic substrate 7-ethoxy-3H-phenoxazin-3-one or resorufin ethoxy ether (Eres) (# 16122) was purchased from Cayman Chemical (Ann Arbor, MI, USA). OptiPlate-384 Black, Black Opaque 384-well Microplates (part no. 6007270, Perkin Elmer, Waltham, MA, USA) were used for all fluorogenic experiments. MilliQ water (18.2 MΩ) (MilliporeSigma, Carlsbad, CA, USA), was used to prepare 0.1 M potassium phosphate buffer (pH 7.4) with potassium phosphate dibasic (0.06958 M) and potassium phosphate monobasic (0.03042 M). OP stocks were prepared at 0.5 mM (10 µM working concentration) in dimethylsulfoxide (DMSO), which were further diluted to 0.05 mM (1 µM final working concentration). Then, 1.2 µL (0.5 or 0.05 mM) of each OP stock was added to 28.8 µL of an enzyme-containing master mix, comprising 2 mg/mL CYP2C9, 0.65 mM NADPH, and 3.3 mM MgCl_2_. The compounds were preincubated with the master mix for 10 or 30 min. An amount of 30 µL of 4 µM Eres was then added to the master mix with OPs in each well after the preincubation time was complete. Fluorescent metabolite formation was determined by measuring the fluorescence (Ex 535, Em 590) with a BioTek Synergy H1 plate reader (BioTek, Santa Clara, CA, USA) for 40 min under the following conditions: speed (normal); delay (100 ms); measurements/read (100/read); read time interval (2 min); height (9.75 mm); and gain (100). Sulfaphenazole was used as a positive control for inhibition of CYP2C9. The slope was calculated as the change in relative fluorescent units over time, and the data were normalized to the average slope of a vehicle control, and the % inhibition was calculated using Equation (1).

(1) $$\%\;inhibition=\frac{Slope\left(Control\right)-Slope\left(OP\right)}{Slope\left(Control\right)}\times 100$$

All data analyses were performed using the Prism 10.4.2 software (GraphPad software Inc., Boston, MA, USA). Experiments were performed with at least three technical replicates per experimental condition, and the reported values are the average of the multiple replicates. Pairwise comparisons were performed using a Mann–Whitney test, with the Holm–Šídák method of multiple comparisons, and a value of 0.05 was used to determine significance.

### 2.3. LC-MS/MS Metabolism

CYP2C9 clearance: CYP2C9 metabolism experiments were performed by BioDuro (Shanghai, China). Recombinant CYP2C9 (15 pmol) in phosphate buffer was preincubated with OPs (1 µM final reaction) at 37 °C for 5 min before addition of NADPH (1 mM final reaction). Ten percent of the reaction was removed and quenched with a 5/10 ng/mL terfenadine/tolbutamide solution in acetonitrile at 0, 5, 30, 60, and 120 min. The samples were centrifuged at 4000 rpm at 4 °C for 15 min, and the supernatant was mixed 1:1 with water for LC-MS/MS analysis with either a Q Trap 4500 or API 4000 (Sciex, Framingham, MA, USA). Diclofenac was used as the positive control CYP2C9 substrate. All LC-MS/MS data are the result of a single biological replicate. The parameters were calculated as follows:

(2) $$\%\;Remaining=\frac{Peak\;area\;ratio\;at\;appointed\;time}{Peak\;area\;ratio\;at\;0\;min}\times 100\%$$

(3) $$t_{1/2\;}=\frac{LN2}{ke}\;;\;{CL}_{int}=\frac{0.69\;}{t_{1/2}}\;\times \frac{Incubation\;volume\;(µL)}{Protein\;amount\;(pmol)}$$

### 2.4. Molecular Docking Analysis

#### 2.4.1. Protein and Ligand Preparation

The crystal structure of human Cytochrome P450 CYP2C9 with bound warfarin (PDB 1OG5) was used for subsequent molecular docking simulations [41]. The structure was imported into the Molecular Operating Environment (MOE) software (v2024.06), where chain A was isolated, and warfarin was removed [42]. Protein preparation was performed using MOE’s QuickPrep function, which applies homology modeling to complete missing residues, adds hydrogens, determines protonation states, and refines the structure using the Amber14:EHT force field [43,44]. The receptor was then converted to pdbqt format using the utility tool prepare_receptor in the ADFR suite [45]. For the ligands, the OP pesticides’ SMILES strings were imported into MOE and prepared using the Wash method, where hydrogens were added, and the 3D coordinates were reconstructed. An energy minimization was performed with the Amber14:EHT forcefield [44]. The prepared compound structures were then converted to pdbqt format using the Python program Meeko (v0.5.0), which supports atom typing and enhanced sampling of macrocycles during docking [46].

#### 2.4.2. Docking Method

Molecular docking calculations were conducted using Autodock Vina version 1.1.2, with the grid space centered at −22.89, 88.38, and 33.046 Å and expanded by 18, 20, and 20 points in the x-, y-, and z-directions, respectively, to encompass the iron heme and nearby residues. The protein was kept rigid during the docking calculations. Before docking the OP pesticides, warfarin was re-docked onto its crystallographic binding site to validate the protocol. Subsequently, each OP pesticide was docked into the receptor at an exhaustive level of 200, generating up to 20 unique poses.

### 2.5. Correlation Analysis

Time-dependent differences in inhibition were calculated from the 1 µM and 10 µM inhibition experiments with preincubation of the substrate for either 10 or 30 min. Similarly, the percentage of the parent remaining after 2 h in the LC-MS/MS experiments was determined. Correlation analyses were performed in GraphPad Prism v10.4.2. We hypothesized that metabolism would only occur if the iron center interacted with the double-bonded oxygen or sulfur, so from the docking calculations, a reactive distance was computed for all poses representing the distance between the P=O for oxons or P=S for thions and the iron atom in the heme. For each OP, the BE of the pose with the shortest reactive distance was used in all subsequent analyses. Additionally, the binding efficiency (BE_eff_), the BE divided by the heavy-atom molecular weight, and the shortest reactive distance were correlated against the time-dependent difference in inhibition or percentage of the parent OP remaining. For each correlation analysis, the R-value was obtained from the equation for the linear fit of the data. Pearson and Spearman rho correlation analyses were performed in GraphPad Prism 10.4.2 using the linear fit correlation module.

## 3. Results

### 3.1. CYP2C9 Activity Is Inhibited by Thions and Oxons

**Figure 1:**
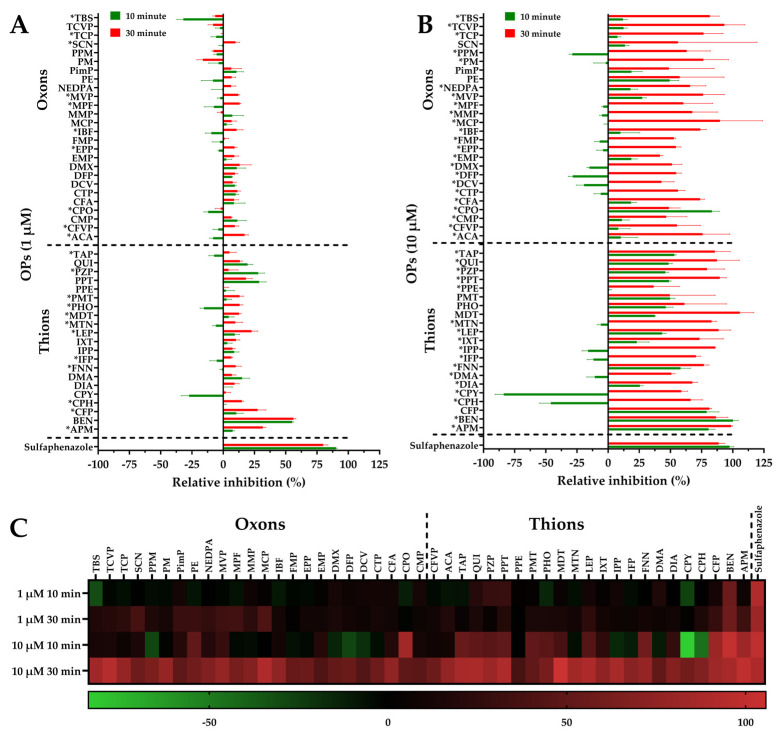
**OP-mediated relative inhibition using pFluor50 fluorogenic assay for CYP2C9 activity.** (**A**) Relative inhibition of CYP2C9 activity by thions and oxons at 1 µM concentration. (**B**) Relative inhibition of CYP2C9 activity by thions and oxons at 10 µM concentration. (**C**) Heatmap showing variations in CYP2C9 activity due to OP inhibition at 1 and 10 µM between 10 and 30 min. The color scale represents percent activation/inhibition from −85 (green; activation) to 105 (red; inhibition). * Represents OP compounds wherein the inhibition of CYP2C9 changes significantly between 10 and 30 min. Sulfaphenazole was used as a positive control for inhibition of CYP2C9. Dashed lines represent segregation of OP compounds as either oxons or thions and the control inhibitor Sulfaphenazole.

We used our previously developed pFluor50 high-throughput fluorogenic assay to determine the inhibition of CYP2C9 activity by the OP compounds using Eres as a substrate and sulfaphenazole as a positive control inhibitor [47]. We performed the activity assay with 47 different OP derivatives to determine if they are inhibitors or activators of CYP2C9. Sulfaphenzole was used as a positive control as it almost completely inhibits CYP2C9 activity at the concentrations used (Figure 1A,B). We determined that out of 21 thions, 18 at 10 µM and 12 at 1 µM exhibited significant time-dependent variations in inhibition when preincubated for 10 or 30 min (Figure 1A,B). Meanwhile, out of 26 oxons, 23 at 10 µM and 10 at 1 µM exhibited significant time-dependent variations in inhibition between 10 and 30 min, respectively (Figure 1A,B). The data are also summarized in a heatmap of the OP-mediated inhibition of CYP2C9 activity (Figure 1C). Overall, we found that several OP pesticides (CPY, PHO, EPP, IBF, MPF, etc.) were activators of CYP2C9 after 10 min of preincubation but became inhibitors after 30 min of preincubation. Furthermore, some OP pesticides, such as BEN and CPO, showed a time-dependent decrease in inhibition. Time-dependent increases in inhibition suggest either mechanism-based OP inhibition of CYP2C9 with a slow second step or, more likely, that the OP is metabolized by CYP2C9, leading to a product/metabolite that is more inhibitory. Decreases in inhibition suggest metabolism with the product/metabolite being less inhibitory toward CYP2C9.

### 3.2. Thions Are Selectively Metabolized by CYP2C9 (Compared with Oxons)

**Figure 2:**
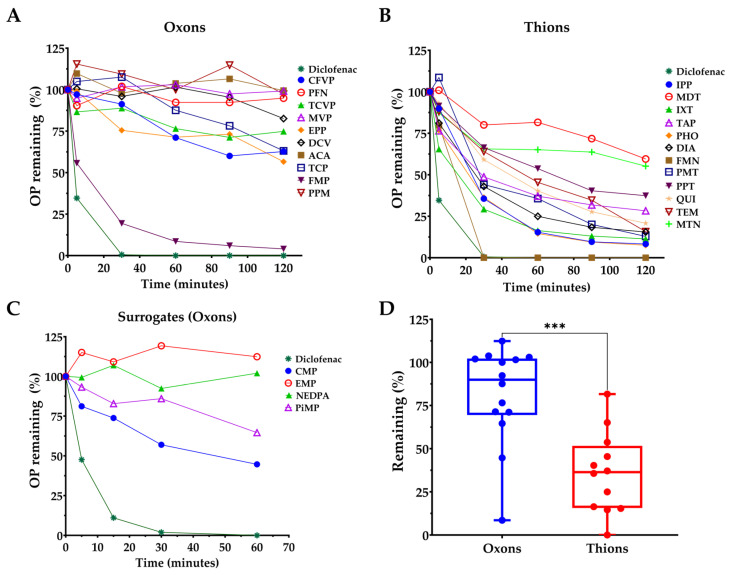
Metabolism of OP compounds by CYP2C9 measured by disappearance of the parent. (**A**) LC-MS/MS degradation of oxons by CYP2C9 after 2 h. (**B**) LC-MS/MS degradation of thions by CYP2C9 after 2 h. (**C**) LC-MS/MS degradation of CWNA surrogates after 1 h. (**D**) CYP2C9-mediated degradation of thions was significantly higher compared with oxons based on mean averages after 2 h. Each dot represents either an oxon (blue) or a thion (red). *** *p* < 0.001.

We analyzed the metabolism of thions and oxons (including CWNA surrogates) as determined by the percentage of the parent OP remaining from LC-MS/MS experiments and determined the intrinsic clearance rate. We observed that all 12 of the thions studied were significantly degraded by CYP2C9 after 2 h, with MDT being metabolized the slowest but still degraded by at least 35% (Figure 2B). FMN was completely degraded within 30 min, while DIA, PHO, IXT, QPS, and TEM were metabolized by ~80% after 2 h (Figure 2B). Among the 10 oxon pesticides, only FMP was metabolized completely (Figure 2A), whereas TCP, TCVP, CFVP, and EPP were metabolized by ~35%. The other oxon pesticides studied were not significantly metabolized within the time studied. The tested CWNA surrogates were only incubated with CYP2C9 for 1 h (Figure 2C); however, CMP (cyclosarin surrogate) and PiMP (soman surrogate) were metabolized by ≥35%. The intrinsic clearance rate calculated for each of the OP compounds is listed in Table 1. Overall, we found that thions (mean 35.85%) were metabolized more readily than oxons (81.38%), with the mean difference being −45.53 ± 10.30 and the confidence interval of the difference being (−66.79 to −24.26) from oxons to thions (Figure 2D).

### 3.3. Molecular Docking Analysis Reveals Correlation Between Binding Energy and Degradation or Time-Dependent Change in Inhibition

**Figure 3:**
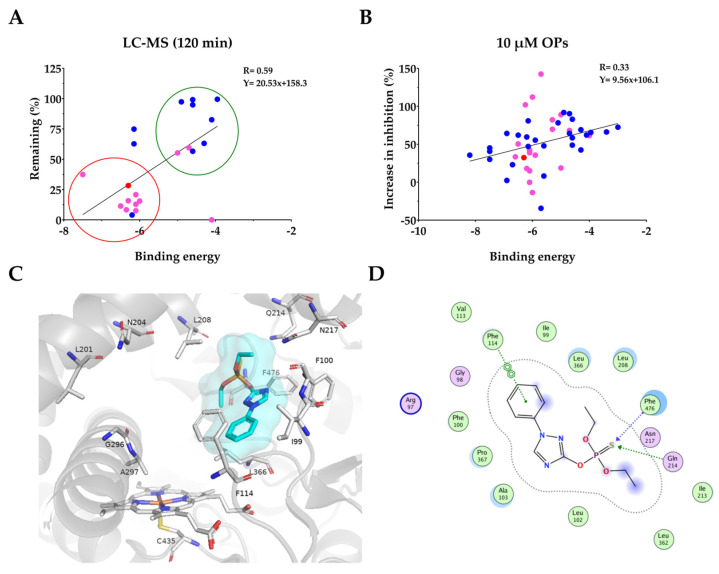
**Correlation analysis between binding energy (kcal/mol) of the pose with the shortest reactive distance (docking) vs. experimental results.** (**A**) Correlation analysis (R = 0.59; *p* < 0.001; Spearman Rho R = 0.51; *p* < 0.01) between the percent of the parent remaining in the LC-MS/MS metabolism experiments after 2 h and the binding energy in the docking experiments. Pink circles represent thions, blue circles represent oxons, and the red circle is TAP. (**B**) Correlation analysis (R = 0.33; *p* < 0.01; Spearman rho R = 0.43; *p* < 0.001) between the time-dependent increase in inhibition (%) in the pFlour50 assay and the binding energy. Pink circles represent thions, blue circles represent oxons, and the red circle is TAP. (**C**) TAP docks into the 1OG5 active site nearly perpendicular to the heme ring. (**D**). Two-dimensional interaction diagram of TAP with 1OG5 amino acids showing the interactions of Phe114 (Π-Π) and Phe476/Gln214 with the sulfur. Residues are colored by type (purple = polar, red = acid, blue = basic, green = greasy), grey dashed outline shows the proximity contour, dashed arrows indicate hydrogen bonds (green = sidechain, blue = backbone), and green aromatic symbols denote Π interactions.

We performed molecular docking using Autodock Vina with the OP compounds studied experimentally (Figures S1 and S2). Docking was performed with PDB 1OG5 as the CYP2C9 receptor, which was selected, as it has one of the highest resolutions and has been used previously for docking analyses [41,48,49,50]. We then performed a correlation analysis of the percentage of the parent OP remaining after 2 h with the computed BE from the pose with the shortest reactive distance. We also explored the correlation of the experimental data with the shortest reactive distance between the OP atom and the iron atom in the heme ring. Additionally, a binding efficiency (BE_eff_) was computed, representing the BE divided by the heavy-atom molecular weight of the OP compound. Notably, we identified that the computed BE of the pose with the shortest reactive distance yielded the best correlation with the experimental data. Figure 3A shows the correlation between the percentage of the parent remaining and the BE for the pose with the shortest reactive distance, with a correlation coefficient of R = 0.59 (*p* < 0.001). This analysis excluded CWNA surrogates as their metabolism was only tested for 1 h. The OP compounds that were metabolized to a greater extent had lower binding energies, while the OP compounds that were not metabolized had higher binding energies. Thus, OP compounds could be clustered into two groups (Figure 3A) except for FMN, PPT, CFVP, and TCVP. We also performed a similar analysis with the degradation data at 1 h (which included the surrogates) but found a much lower correlation (R = 0.40; *p* < 0.05) (Figure S3A). The correlation between the degradation and the BE_eff_ of the pose with the shortest reactive distance for both 2 h and 1 h data can be seen in Figure S3B–E. TAP was the OP that demonstrated the strongest correlation with the in silico binding energy compared with the LC-MS/MS degradation and inhibition potential (Figure 3C,D). TAP was found to interact with both polar and nonpolar as well as aliphatic and aromatic residues. It interacted with CYP2C9 via Phe114 (Π-Π) and Phe476/Gln214 and the sulfur group, while Phe100 was another aromatic amino acid in the binding pocket. It also interacted with polar amino acids such as Arg97, Asn 217, and Gln214, while non-polar residues involved in the interaction included Val113, Ile99, Leu366, Leu208, Ile205, Leu362, Leu102, Ala103, Pro367, and Gly98.

Next, we performed a correlation analysis between the time-dependent change in inhibition (between 10 and 30 min of preincubation) at the 10 µM OP concentration with the computed binding energy of the pose with the shortest reactive distance. We, again, obtained a positive correlation with a correlation coefficient of R = 0.33 (*p* < 0.01) (Figure 3B). Unlike the LC-MS/MS analysis, which was only performed for 22 of the OP compounds, this analysis included all 47 OP derivatives. When we analyzed the correlation between the time-dependent change in inhibition with the calculated binding energy for just the oxons, we obtained R = 0.43 (*p* < 0.01) (Figure S4A), whereas a similar analysis with only the thions resulted in R = 0.22 (P:NS). Similar analyses were also performed for the 1 µM OP time-dependent change in inhibition and the calculated binding energy (R = 0.06; P:NS) (Figure S3C). Similarly, we performed a correlation analysis for the time-dependent change in inhibition at the 10 µM OP concentration with the shortest reactive distance between the OP and the heme ring center (R = 0.13; P:NS) and BE_eff_ (R = 0.08; P:NS) (Figure S3D,E). We then correlated the individual inhibition percentage at a particular time (10 or 30 min) and concentration of OP (1 or 10 µM) with the BE but did not observe a strong positive correlation for any condition (Figure S5A–D).

We further compared the 2D interactions between the CWNA surrogates and CYP2C9. CMP, PiMP, and NEDPA did not interact in the same way with CYP2C9 compared with the corresponding CWNAs cyclosarin, soman, and tabun, while EMP could be predictive of VX metabolism, as the interactions were similar to the amino acids Phe114, Ala103, Leu366, Leu 102, Leu208, Ile213, Asn217, Gln214, Phe476, Ile99, Arg97, Gly98, Val113, and Pro367, forming a common binding pocket (Figure S6A–H). This analysis showed that due to the presence of the large coumarin group, the surrogates have very different binding interactions, and this increase in size likely results in changes to the binding site. However, VX has a larger leaving group compared with the other CWNAs, so when it is replaced with coumarin in the surrogate, the binding interactions with CYP2C9 remained unchanged. Nonetheless, the R-values of 0.59, 0.33, and 0.43 demonstrate that the docking studies may have value in predicting OP metabolism.

## 4. Discussion

OP poisoning is among the leading methods of suicide worldwide, accounting for approximately 15% of all suicide-related deaths (150,000–200,000 globally) [2,3,4]. Beyond fatalities, the widespread use of OP pesticides in agriculture frequently exposes workers and results in residues on produce such as fruits and vegetables, contributing to chronic OP exposure in a large portion of the population [8,9,10]. Existing treatments for OP poisoning remain insufficient, especially regarding effects on the CNS. As a result, gaining a deeper understanding of how OP compounds are metabolized is crucial.

We, along with others, have previously demonstrated that human liver microsomes (HLMs) can metabolize many OP compounds using targeted LC-MS/MS and NMR-based metabolomics [19,51,52,53,54]. However, HLMs contain many metabolizing enzymes, including CYPs, which are naturally occurring bioscavenging enzymes that perform xenobiotic metabolism in the human body [55]. We now studied the metabolism of OP compounds by CYP2C9 using LC-MS/MS to determine the degradation of 26 OP derivatives, including 4 CWNA surrogates.

We found that thions were more readily metabolized compared with oxons (Figure 2D). Thions are generally converted to the active metabolite oxon through oxidation of the phosphorus-bound sulfur to the corresponding oxygen [56,57]. CYP2C9 has previously been shown to desulfurate thions, such as diazinon [24], dimethoate [25], disulfoton [26], fenthion [27], malathion [28], and ethyl parathion [29]. Here, we extend these findings to additional OP pesticides while also studying the metabolism of oxons. Particularly, our work confirms and extends the previously demonstrated metabolism of diazinon and malathion by CYP2C9 (Figure 2B). Correlation analysis between the percentage of parent OP remaining after 2 h with the computed binding energy (BE) of the pose with the shortest reactive distance showed a correlation coefficient of R = 0.59 (*p* < 0.001), suggesting that tighter binding leads to greater metabolism. CFVP, TCVP, and FON were outliers, which may be due to the formation of secondary metabolites that inhibit further metabolism by CYP2C9. On average, the BE for thions showed more favorable binding compared with the oxons. However, the BE (−5.7 kcal/mol) of the thion CPY and its corresponding oxon CPO were very similar, suggesting that moieties other than just the sulfur versus oxygen on the phosphorus of the OP compound may contribute to the interaction with CYP2C9.

We previously developed a fluorogenic assay for CYP2C9 using Eres as a substrate, pFlour50 [47]. We further showed that sulfaphenazole inhibits CYP2C9 in a dose-dependent but time-independent manner. Using this previously developed method, we determined the time-dependent CYP2C9 activation and/or inhibition by OP compounds. We performed the inhibition studies at two different concentrations (1 and 10 µM) and two different preincubation times (10 and 30 min) to study the time- and dose-dependence of the inhibition. We found that at the 10 µM OP concentration, there was a significant change in CYP2C9 inhibition for a large proportion of the OP compounds studied, indicating either mechanism-based inhibition or metabolism by CYP2C9 to secondary metabolites with differences in binding affinity. Particularly, some OPs, such as CPY, PHO, EPP, IBF, MPF, etc., were activators after 10 min but became inhibitors at 30 min, while others demonstrated a decrease in inhibition, like PE, BEN, and CPO, suggesting that these compounds may be further metabolized to secondary metabolites that have lower binding affinity compared with the original OPs. Comparing the LC-MS/MS metabolism data with the pFlour50 inhibition data, we observed several key findings. FMP was the only oxon metabolized completely, and the inhibition data also showed more than a 50% increase in inhibition. Among the thions, IPP, IXT, PHO, DIA, PMT, and QUI were metabolized by 80% or more and demonstrated a more than 50% increase in inhibition at the 10 µM concentration in the pFlour50 assay (Figure 1 and Figure 2). These data suggest that the increased metabolism and the increase in inhibition may be linked, implying that the inhibition may be the result of the secondary metabolites that are produced. Indeed, it has previously been shown that the metabolite produced by initial CYP3A4 or CYP3A5 metabolism of lapatinib inhibits the enzymes [57,58,59,60]. However, the methods used in the current study do not identify the metabolites produced nor directly explore their inhibitory potential, so the underlying mechanisms are speculative. Future studies that identify the metabolites produced and directly explore the inhibition of those metabolites should be investigated.

This study and others like it could perhaps be used for experimental validation of online prediction software such as SwissADME, Deep-PK, and ADMETlab3 ([61,62,63]), which are useful open-source tools to predict inhibition for CYP450s such as CYP2C9. For example, these software programs do not predict that MVP will inhibit CYP2C9. Nonetheless, we found MVP was an inhibitor of CYP2C9; thus, using the pFlour50 assay to determine the inhibition of CYP2C9 as well as other CYPs could help to validate the predictions of bioinformatics software and help refine the algorithms for prediction of inhibition. Overall, these results suggest potential mechanisms that may be further investigated in the future. Furthermore, these changes were not as prevalent at the 1 µM OP concentration, suggesting that the affinity of CYP2C9 for the OP compounds may be rather low, perhaps due to their small size, which may only partially fill the enzyme active site.

Docking analysis revealed that the time-dependent change in inhibition at 10 µM was positively correlated with the BE (R = 0.33; *p* < 0.01) of the pose with the shortest reactive distance. The majority of the oxons studied fit well to the correlation curve, except for CPY and CPO. CPY showed a nearly 140% increase in inhibition, whereas CPO showed a decrease in inhibition with time, suggesting that it is metabolized to a product with lesser inhibition of CYP2C9. Interestingly, CPY (the corresponding thion of the oxon CPO) inhibition after the 10 min pre-incubation demonstrated it was an activator (~84%), whereas after the 30 min pre-incubation, it was an inhibitor ~59%. CPO inhibition at the same time was ~83%. This suggests that CPY may be metabolized after 30 min to a mix of CPY and CPO, resulting in greater inhibition of CYP2C9. If this oxon–thion pair is removed from the correlation analysis, the correlation coefficient would be R = 0.39. For the thions studied, there were multiple OPs, such as PHO, PMT, and IPP, which demonstrated significant metabolism in the LC-MS/MS studies and also showed time-dependent increases in inhibition, suggesting that these compounds are metabolized into secondary metabolites that are more inhibitory toward CYP2C9. Similarly, this might be the reason for the variations we observed with other thions such as APM, BEN, CPH, CPY, CFP, and fenthion. The data showed that thions had greater variability in inhibition with time (both increased and decreased) compared with oxons. This could be due to the conversion of thions to the corresponding oxons, as the LC-MS/MS data showed metabolism of the majority of thions. Overall, although the positive correlation observed (R = 0.59; *p* < 0.001) is modest, it indicates that it might be possible to predict OP metabolism based on the computed BE. Repeating these experiments with additional OP compounds in the future might improve the correlation values. Additionally, future MD simulations or perhaps rescoring using a more rigorous algorithm, such as Molecular Mechanics Poisson–Boltzmann Surface Area (MM-PBSA), may provide increased correlations compared with the static docking and associated binding energy performed here. Furthermore, multiple regression models with additional parameters (for example, combining LogP, MW, and shortest distance along with BE) may also allow for more accurate predictions, perhaps allowing for the development of an AI/ML model for predicting OP metabolism.

The CWNA surrogate data analysis demonstrated that CMP (a surrogate of cyclosarin) and PiMP (a soman surrogate) were both metabolized by CYP2C9 after 1 h. Furthermore, CMP had the largest van der Waals radii, which may have contributed to stronger interactions leading to its calculated BE, which was the lowest among the surrogates studied. Thus, comparing the binding pose with the shortest reactive distance for CMP versus cyclosarin, PiMP versus soman, and NEDPA versus tabun showed multiple differences, suggesting that these surrogates might not represent the metabolism of CWNAs by CYP2C9 (Figure S6) and, furthermore, that CYP2C9 may not metabolize the authentic CWNAs. However, VX and EMP (a surrogate of VX) bound in the same binding pocket and had very similar interactions with the enzyme, suggesting that EMP is likely predictive of VX, and indicating that VX is likely not metabolized by CYP2C9 since EMP was also not degraded even after one hour (Figure S6E,F). We used CWNA surrogates in this paper, which are vastly different in size and, therefore, likely interact with different residues in static docking analysis. Additionally, the LC-MS/MS metabolism studies for the surrogates were for 1 h (rather than 2 h); therefore, it must be noted that the CWNAs could actually be metabolized by CYP2C9, and this should be explored in the future.

## 5. Conclusions

In conclusion, although CYP2C9 is likely responsible for the metabolism of the majority of thion OP pesticides, it likely is not involved in the metabolism of CWNAs. In this study, we were able to demonstrate a moderate correlation, but perhaps with additional compounds, a stronger correlation might be observed. Furthermore, we only performed docking on the OP compounds, but this may be improved in the future by using more robust molecular dynamics simulations for the degraded OPs. The lower correlation between BE and time-dependent changes in inhibition, especially for the thions, suggests that the metabolism observed in the degradation studies may affect the inhibition studies, so perhaps a decrease in the time of incubation to 20 min may provide better correlation for the thions.

Recently, an AI/ML model was used to predict 40,000 new OP structures from VX, many of which were predicted to be more toxic (based on a predicted increase in inhibition of AChE) than the starting CWNA structure VX [30,31]. So, to investigate the metabolism of all of these structures with CYPs, we need to develop better AI/ML predictive models using CYPs. While experimental analysis of all these theoretical structures is not possible, the experiments and computational analysis contained in the current study suggest that through analysis of the susceptibility of a subset of OP compounds to degradation along with the computed BE of the pose with the shortest reactive distance, it may be possible to develop AI/ML-based models to predict the metabolism of current and emerging CWNAs. In the future, we will explore similar analyses of OPs with other CYPs to determine if these results are specific to CYP2C9 or are more broadly applicable to all the relevant human CYPs.

## Figures and Tables

**Table 1:** Half-life and intrinsic clearance of OP pesticides by CYP2C9, as measured by disappearance of the parent. The half-lives for compounds with clearance of less than 0.07 µL/min/pmol of CYP in 60 min or 0.04 µL/min/pmol of CYP in 120 min are, therefore, reported as greater than the longest calculable half-life for that time (186.4 min or 372.8 min, respectively).

Compound	t_1/2_ (min)	CL_int_ (µL/min/pmol CYP)
***Control***		
Diclofenac	<5	>2.8
***Oxons***		
PFN, DCV, ACA, PPM, MVP	>372.8	<0.04
EMP and NEDPA	>186.4	<0.07
CFVP	153.3	0.1
TCVP	214.3	0.4
TCP	172.2	0.1
EPP	164.9	0.1
CMP	54.85	0.25
PiMP	105.46	0.13
FMP	27.1	0.5
***Thions***		
IPP	31.3	0.4
MDT	171.6	0.1
PHO	31.2	0.4
IXT	31.5	0.4
TAP	57.3	0.2
DIA	44.2	0.3
FON	15.3	0.9
PHT	39.8	0.3
PPT	82.6	0.2
TEM	49.4	0.3
QPS	52.2	0.3
MTN	52.4	0.3

## Data Availability

The original contributions presented in this study are included in this article/the Supplementary Materials. Further inquiries can be directed to the corresponding author.

## Supplementary Materials

The following supporting information can be downloaded at https://www.mdpi.com/article/10.3390/jox16010001/s1. Figure S1: Structures of the oxons used in experiments and docking analysis; Figure S2: Structures of the thions used in experiments and docking analysis; Figure S3: Correlation between LC-MS/MS data with docking parameters; Figure S4: Correlation between time- and dose-dependent inhibition data between 10 and 30 min with docking parameters based on thions and oxons; Figure S5: Correlation between time-specific inhibition data with docking parameters; Figure S6: Two-dimensional docking interaction diagram of OPNA and their surrogates; Table S1: Table of general properties of the OPs.

## Abbreviations

The following abbreviations were used in this manuscript:

AChE	Acetylcholinesterase
ACA	Acephate
Å	Angstroms
APM	Azinphos-methyl
BNS	Bensulide
BE	Binding energy
BEeff	Binding efficiency
CNS	Central nervous system
CWNA	Chemical warfare nerve agent
CFVP	Chlorfenvinphos
CPH	Chlorphoxim
CPY	Chlorpyrifos
CPO	Chlorpyrifos oxon
CTP	Crotoxyphos
CFA	Crufomate
CFP	Cyanofenphos
CYP	Cytochrome P450
°C	Degrees Celsius
DFP	Diisopropyl fluorophosphate
DIZ	Diazinon
DCV	Dichlorvos
DEP	Diethylparaoxon
DMX	Dimefox
DMA	Dimethoate
DMP	Dimethyl paraoxon
DMSO	Dimethylsulfoxide
Em	Emission
EPP	Ethoprophos
Eres	7-ethoxy-3H-phenoxazin-3-one or resorufin ethoxy ether
Ex	Excitation
EHT	Extended Hückel theory
FMP	Fenamiphos
FNN	Fenthion
FON	Formothion
HBA	Hydrogen bond acceptor
HBD	Hydrogen bond donor
Inc.	Incorporated
IFP	Iodofenphos
IBF	Iprobenfos
IPP	Isofenphos
IXT	Isoxathion
LPS	Leptophos
LC	Liquid chromatography
MTN	Malathion
MS	Mass spectrometry
LD_50_	Median lethal dose
MPF	Mephosfolan
MMP	Methamidophos
MDT	Methidathion
MVP	Mevinphos
µl	Microliter
µM	Micromolar
mg	Milligram
ml	Milliliter
mm	Millimeter
mM	Millimolar
ms	Millisecond
M	Molar
MM-PBSA	Molecular Mechanics Poisson–Boltzmann Surface Area
MOE	Molecular operating environment
MW	Molecular weight
MCP	Monocrotophos
ng	Nanograms
NADPH	β-nicotinamide adenine dinucleotide phosphate
NS	Not Significant
OP	Organophosphorus Pesticide
PHO	Phosalone
PFN	Phosfolan
PHT	Phosmet
PPM	Phosphamidon
pmol	Picomoles
PPE	Pirimiphos-ethyl
PSA	Polar surface area
PDB	Protein Data Bank
PZP	Pyrazophos
PPT	Pyridaphenthion
QNP	Quinalphos
rpm	Revolutions per minute
SCN	Schradan
SMILES	Simplified Molecular Input Line Entry System
Sul	Sulfaphenazole
TEM	Temephos
TCVP	Tetrachlorvinphos
TAP	Triazophos
TBS	Tribufos
TCP	Trichlorphon
