# Supplementary material for: Human CYP2C9 Metabolism of Organophosphorus Pesticides and Nerve Agent Surrogates
Source: J Xenobiot. 2025 Dec 19;16(1):1. doi: 10.3390/jox16010001 (PMC12821593; doi:10.3390/jox16010001)
Supplement: Supplementary file 1 [file jox-16-00001-s001.zip › jox-3997819-supplementary.pdf]

Journal of Xenobiotics

MDPI

Supplementary Materials: Human CYP2C9 Metabolism of Organophosphorus Pesticides and Nerve Agent Surrogates

Pratik Shriwas, Abigail M. Noonchester, Andre Revnew, Thomas R. Lane, Christopher M. Hadad, Sean Ekins and Craig A. McElroy

Figure S1. Structures of the oxons used in experiments and docking analysis

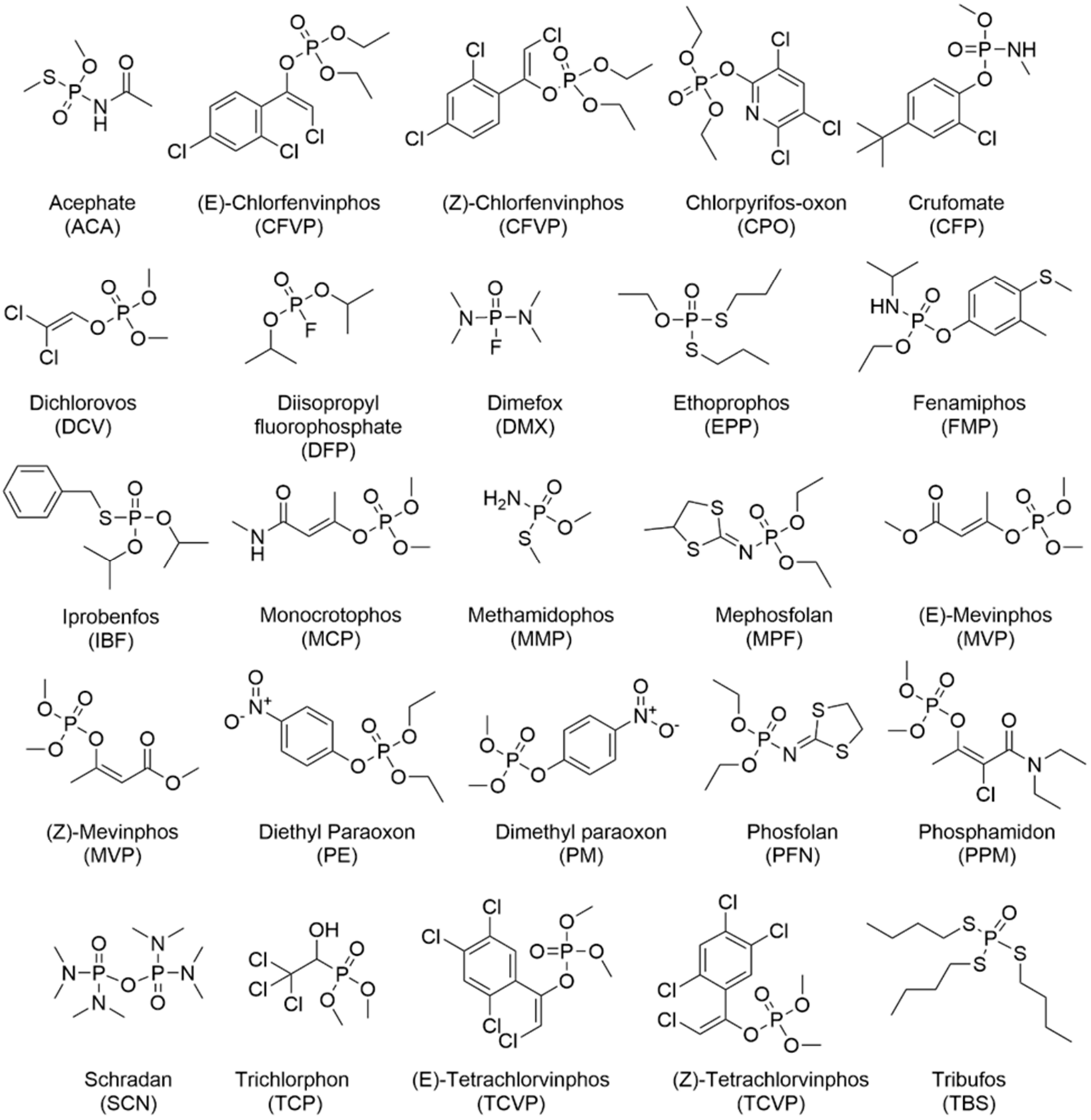

Structures of the oxons used in experiments and docking analysis

 https://doi.org/10.3390/jox16010001

Figure S2. Structures of the thions used in experiments and docking analysis

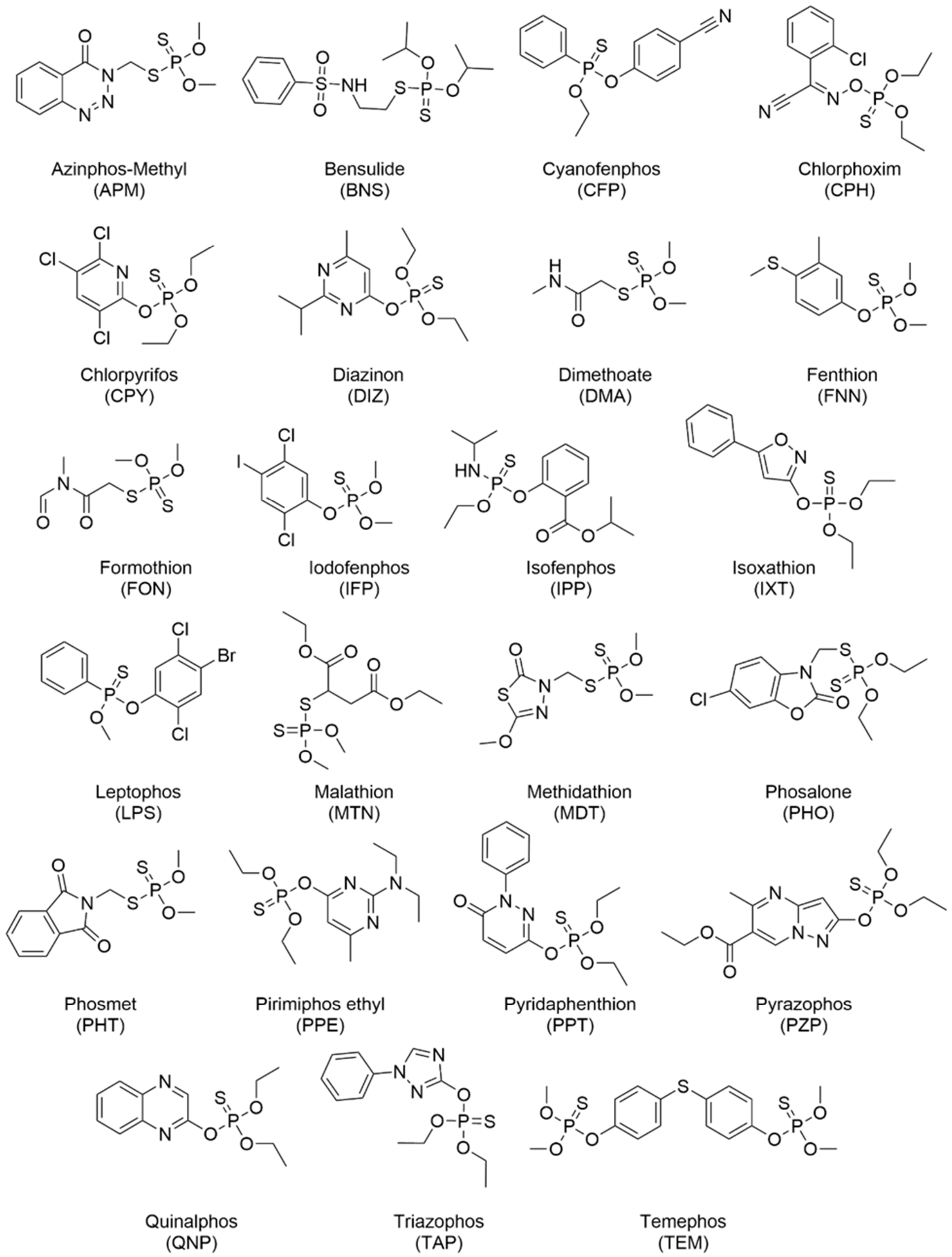

Structures of the thions used in experiments and docking analysis

Figure S3. Correlation between LC-MS/MS data with docking parameters

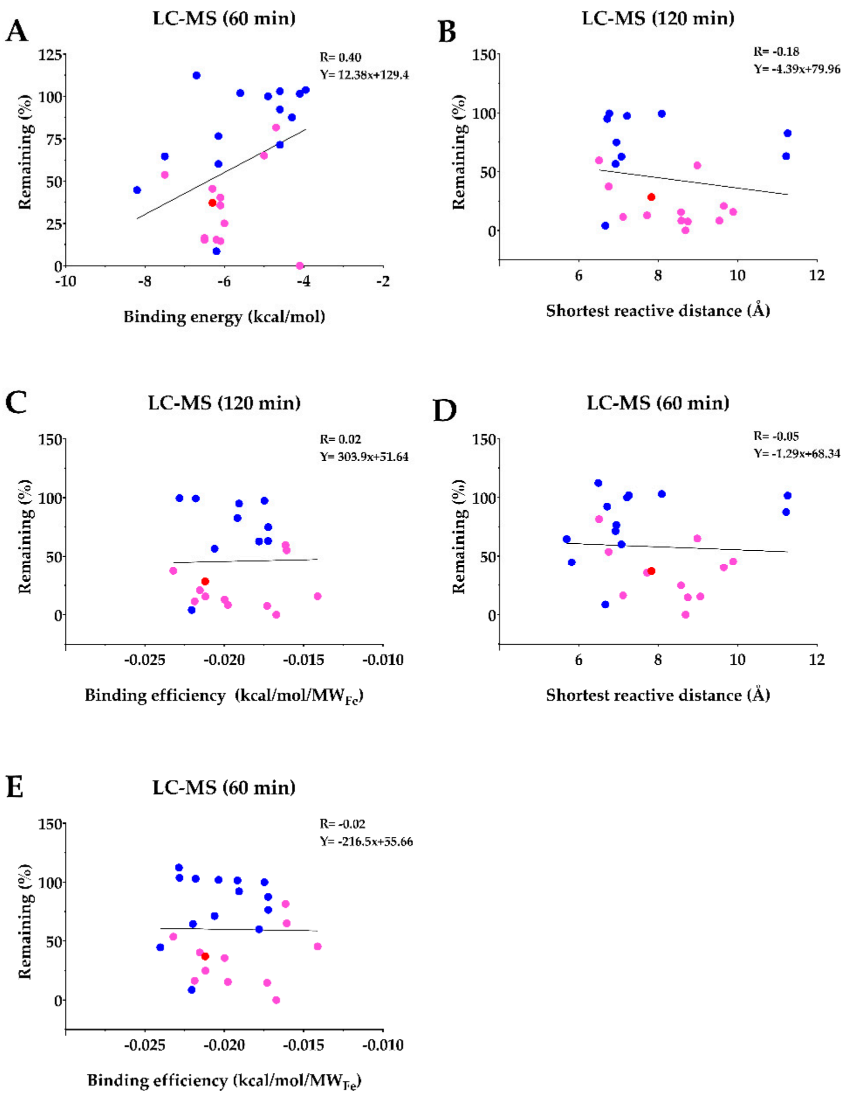

A. Correlation analysis between metabolite remaining in LC-MS/MS metabolism (1 hour) and Binding energy. B. Correlation analysis between metabolite remaining in LC-MS/MS metabolism (2 hours) and shortest reactive distance. C. Correlation analysis between metabolite remaining in LC-MS/MS metabolism (1 hour) and Binding efficiency. D. Correlation analysis between metabolite remaining in LC-MS/MS metabolism (1 hour) and shortest reactive distance. E. Correlation analysis between metabolite remaining in LC-MS/MS metabolism (1 hour) and Binding efficiency. Diclofenac was used as a control for CYP2C9 metabolism. Dot color: Pink: thion; Blue: oxon: Red: TAP.

Figure S4. Correlation between time and dose dependent inhibition data between 10 and 30 minutes with docking parameters based on thions and oxons.

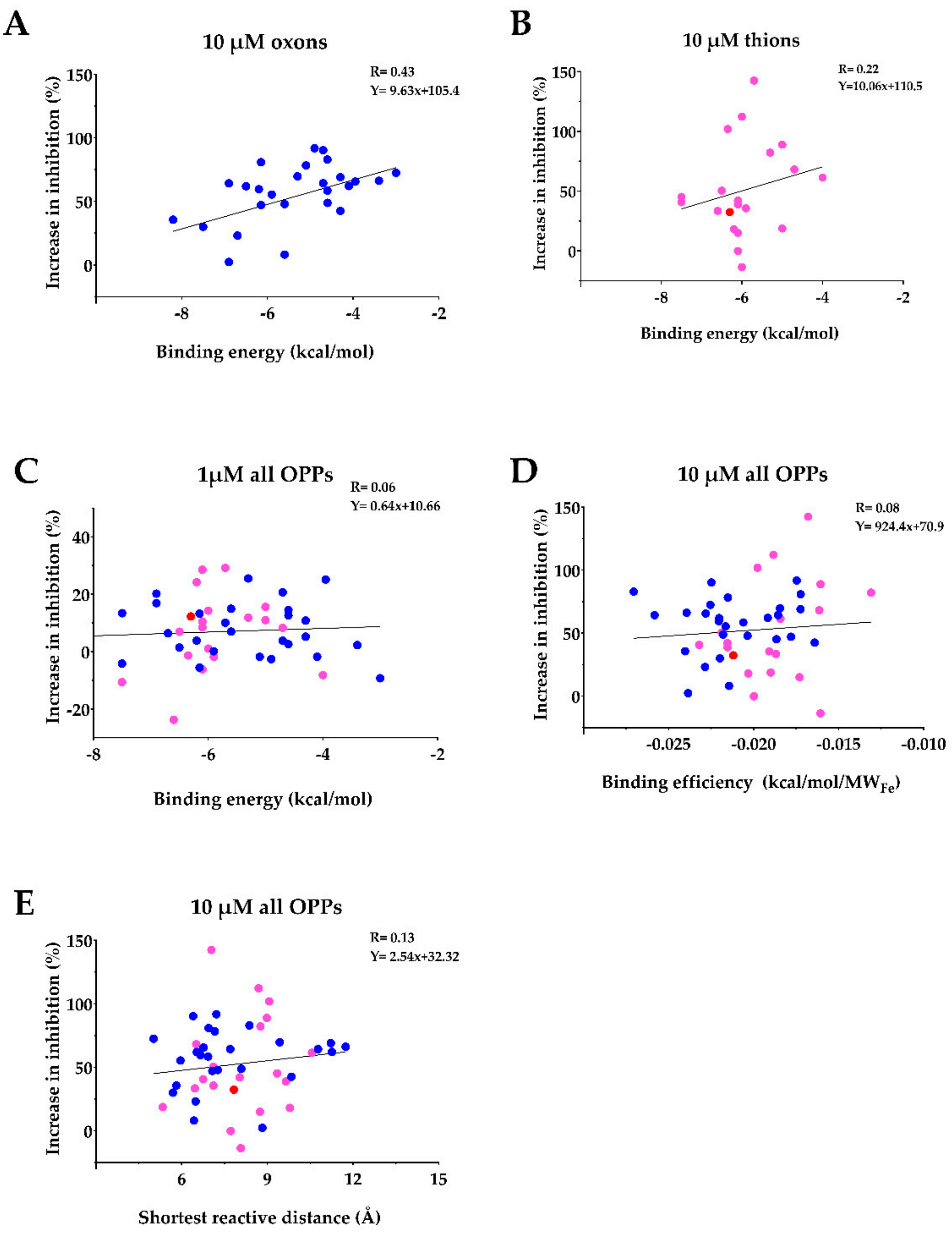

A. Correlation analysis between inhibition data and Binding energy for oxons at 10 μM concentration. B. Correlation analysis between inhibition data and Binding energy for thions at 10 μM concentration. C. Correlation analysis between inhibition data and Binding energy at 1 μM OP concentration. D. Correlation analysis between inhibition data and Binding efficiency at 1 μM concentration. E. Correlation analysis between inhibition data and shortest reactive distance at 1 μM concentration. Sulfaphenazole was used as a control for CYP2C9 metabolism. Dot color: Pink: thion; Blue: oxon; Red: TAP.

Figure S5. Correlation between time specific inhibition data with docking parameters

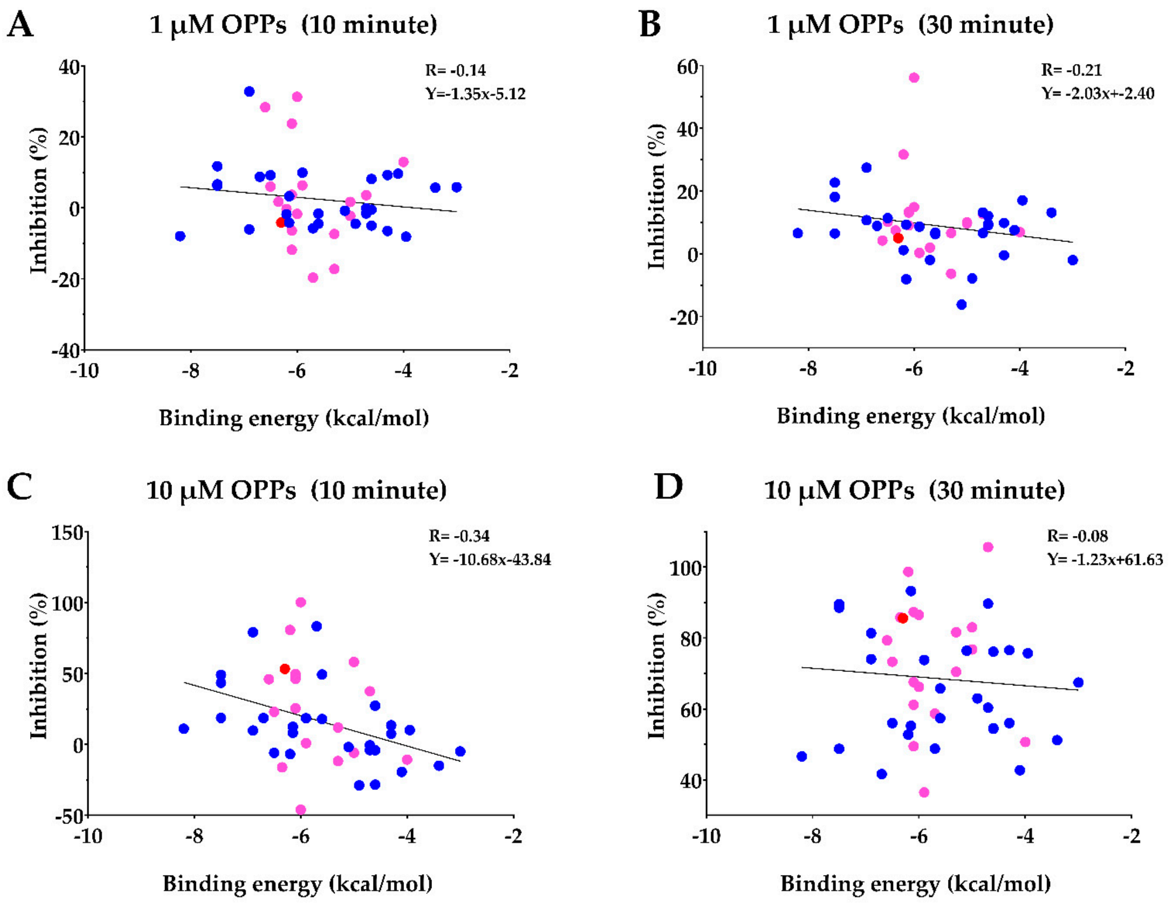

A. Correlation analysis between inhibition data and binding energy for all OPs at 1 μM concentration at 10 minutes. B. Correlation analysis between inhibition data and binding energy for all OPs at 1 μM concentration at 30 minutes. C. Correlation analysis between inhibition data and binding energy for all OPs at 10 μM concentration at 10 minutes. D. Correlation analysis between inhibition data and binding energy for all OPs at 10 μM concentration at 30 minutes. Sulfaphenazole was used as a control for CYP2C9 metabolism. Dot color: Pink: thion; Blue: oxon; Red: TAP.

Figure S6. 2D docking interaction diagram of OPNA and their surrogates

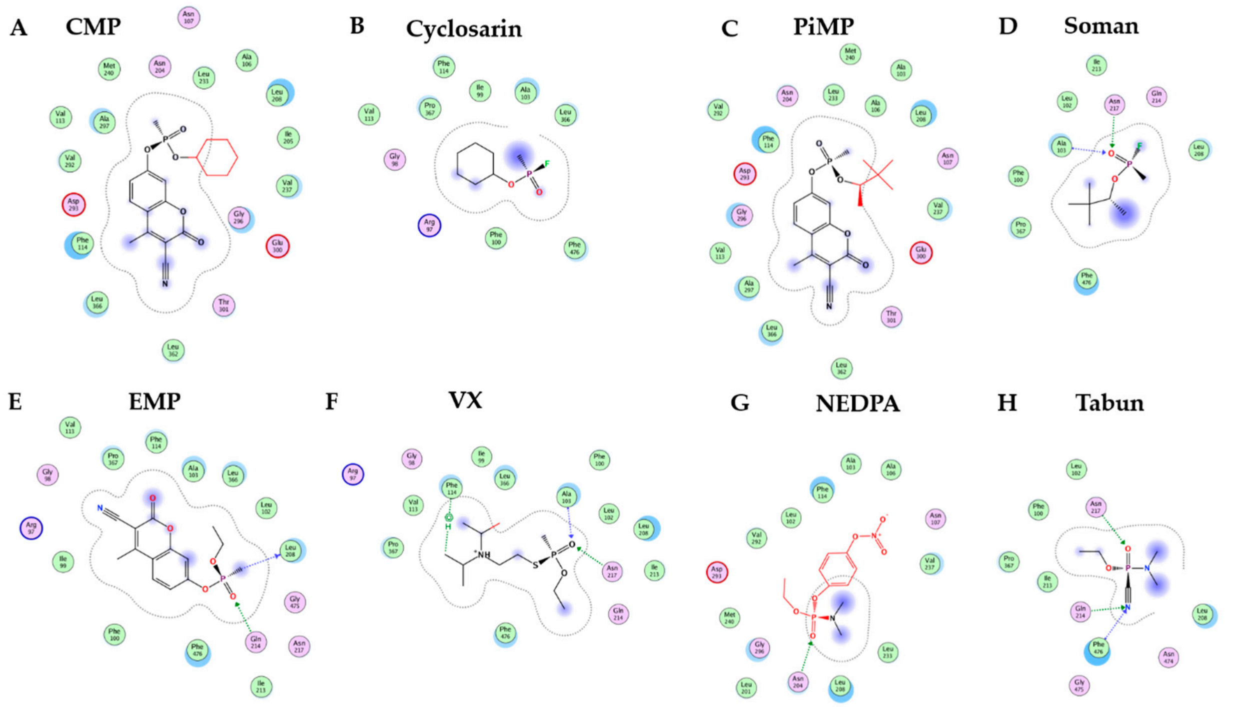

A. 2D interaction diagram of CMP with Phe114, Leu362, Gly296 being closely interacting residues. B. 2D interaction diagram of Cyclosarin with Gly98, Arg97, Phe 100 being closely interacting residues. C. 2D interaction diagram of PiMP with Ala297, Leu366 and Thr301 being closely interacting residues. D. 2D interaction diagram of Soman with Asp217 and Ala 103 forming hydrogen bonds. E. 2D interaction diagram of EMP with Leu208 and Gln214 forming hydrogen bonds. F. 2D interaction diagram of VX with Ala103 and Asn217 forming hydrogen bonds. G. 2D interaction diagram of NEDPA with Asn204 forming hydrogen bonds. H. 2D interaction diagram of Tabun with ASN217, Gln214 and Phe476 forming hydrogen bonds.

Table S1: Table of general properties of the OPs. (LD_{50} – median lethal dose; HBA – hydrogen bond acceptor; HBD – hydrogen bond donor; LogP – partition coefficient; PSA – polar surface area)

OP	Abbreviation	MW	LD_{50} (μM)	HBA	HBD	LogP	PSA	Rotatable bonds
Acephate	ACA	183.17	163.88[32, 33]	2	1	-0.0006	55.4	4
Azinphos-methyl	APM	317.32	0.90[32, 33]	3	0	2.72	63.49	5
Bensulide	BNS	397.51	21.58[32, 33]	2	1	0	64.63	10
Chlorfenvinphos	CFVP	359.57	1.06[32, 33]	1	0	3.4575	44.76	7
Chlorphoxim	CPH	332.74	238.66[32, 33]	2	0	4.6882	63.84	8
Chlorpyrifos	CPY	350.59	5.98[32, 33]	1	0	4.9668	40.05	6
Chlorpyrifos Oxon	CPO	334.50	6.27[32, 33]	2	0	4.2326	57.12	6
Cyclosarin surrogate	CMP	361.33	0.32[38, 39]	3	0	3.016	85.62	5
Crotoxyphos	CTP	314.27	15.91[32, 33]	2	0	2.3893	71.06	8
Crufomate	CFA	291.71	103.45[32, 33]	1	1	3.532	47.56	5
Cyanofenphos	CFP	303.30	4.50[32, 33]	1	0	4.4521	42.25	6
Diazinon	DIZ	304.35	3.32[32, 33]	2	0	5.0438	52.41	7
Dichlorvos	DCV	220.98	11.50[32, 33]	1	0	1.8444	44.76	4
Diethyl paraoxon	DEP	275.19	0.35[32, 33]	2	0	1.697	96.57	7
Diisopropyl fluorophosphate	DFP	184.15	1.04[32, 33]	1	0	2.2165	35.53	4
Dimefox	DMX	154.13	0.21[32, 33]	1	0	0.5649	23.55	2
Dimethoate	DMA	229.26	33.95[32, 33]	1	1	0.445	47.56	6
Dimethyl paraoxon	DMP	247.14	0.26[32, 33]	2	0	0.975	96.57	5
VX simulant	EMP	307.24	0.38[34, 35]	3	0	1.7734	85.62	5
Ethoprophos	EPP	242.34	5.24[32, 33]	1	0	3.2058	26.3	8
Fenamiphos	FMP	303.36	0.63[32, 33]	1	1	3.401	47.56	7
Fenthion	FNN	278.33	3.64[32, 33]	0	0	4.09	27.69	5
Formothion	FON	257.30	30.86[32, 33]	2	0	0.4522	55.84	7
Iodofenphos	IFP	413.00	179.21[32, 33]	0	0	5.16	27.69	4
Iprobenfos	IBF	288.34	74.91[32, 33]	1	0	4.1856	35.53	7
Isofenphos	IPP	345.39	2.58[32, 33]	1	1	4.053	56.79	9
Isoxathion	IXT	313.31	11.36[32, 33]	2	0	4.3074	49.28	7
Leptophos	LPS	412.06	3.32[32, 33]	0	0	5.9015	18.46	4
Malathion	MTN	330.36	170.96[32, 33]	2	0	1.8403	71.06	11
Mephosfolan	MPF	269.30	31.85[32, 33]	1	0	2.9563	47.89	5
Methamidophos	MMP	141.13	6.75[32, 33]	1	1	-0.66	52.32	2
Methidathion	MDT	302.33	2.63[32, 33]	3	0	3.2045	60.36	6
Mevinphos	MVP	224.15	0.50[32, 33]	2	0	0.1996	71.06	6
Monocrotophos	MCP	223.20	1.99[32, 33]	2	1	999.4683	73.86	6
Tabun surrogate	NEDPA	274.21	27.52[36, 37]	2	0	1.699	90.58	6
Phosalone	PHO	367.81	10.36[32, 33]	1	0	4.4271	48	7

OP	Abbreviation	MW	LD_{50} (μM)	HBA	HBD	LogP	PSA	Rotatable bonds
Phosfolan	PFN	255.30	1.11[32, 33]	1	0	2.5432	47.89	5
Phosmet	PHT	317.32	11.31[32, 33]	2	0	2.805	55.84	5
Phosphamidon	PPM	299.69	0.74[32, 33]	2	0	0.3829	65.07	8
Soman surrogate	PiMP	363.50	0.97[34, 38]	3	0	3.5634	85.62	6
Pirimiphos ethyl	PPE	333.39	13.34[32, 33]	3	0	4.4999	55.65	9
Pyrazophos	PZP	373.36	12.85[32, 33]	4	0	3.4558	81.95	9
Pyridaphenthion	PPT	340.33	71.78[32, 33]	2	0	1.836	60.36	7
Quinalphos	QNP	298.30	7.56[32, 33]	2	0	3.5306	52.41	6
Schradan	SCN	286.25	0.56[32, 33]	2	0	0.716	56.33	6
Temephos	TEM	466.50	68.09[32, 33]	0	0	5.6665	55.38	10
Tetrachlorvinphos	TCVP	365.95	347.20[32, 33]	1	0	4.8974	44.76	5
Triazophos	TAP	313.31	6.69[32, 33]	3	0	3.781	55.65	7
Tribufos	TBS	314.50	3.12[32, 33]	1	0	5.2066	17.07	12
Tricholphon	TCP	257.44	26.16[32, 33]	2	1	0.473333	55.76	4

References: Kim et. al., 2021 [32]; Lewis et. al., 2016 [33]; Misik et. al., 2015 [34]; Amitai et. al., 2006 [35]; Meek et. al., 2012 [36]; Sivam et. al., 1984 [37]; Gupta et. al., 2011 [38]; Amitai et. al., 2007 [39]; Backus et. al., 1982 [40].
